# LncRNA GAS6-AS2 regulates vascular smooth muscle cell senescence through the miR-138-5p/AKT1 axis and serves as a diagnostic and prognostic marker for atherosclerosis

**DOI:** 10.1186/s41065-026-00650-5

**Published:** 2026-02-04

**Authors:** Wei Dong, Zhencheng Li, Kai Xie, Xiaowen Lai, Zhaochuan Luo, Yun Qiu, Huan Pu, Ying Zhang

**Affiliations:** 1 Three Departments of Cardiology, The Third Hospital of Shijiazhuang, Shijiazhuang, 050011 China; 2 Guangzhou Medical University, Guangzhou, 511436 China; 3 Department of Vascular Surgery, The First Affiliated Hospital of Guangzhou Medical University, Guangzhou, 511436 China; 4 Cardiovascular Medicine Department, Guanghan People’s Hospital, Deyang, Sichuan 618300 China; 5 Department of Cardiovascular Medicine II, Affiliated Hospital of Shaanxi University of Chinese Medicine, Deputy No.2, Weiyang West Road, Qindu District, Xianyang, 712000 China

**Keywords:** Atherosclerosis, GAS6-AS2, miR-138-5p, AKT1, Senescence

## Abstract

**Background:**

Atherosclerosis (AS) is a leading cause of cardiovascular-related death worldwide. The role and regulatory mechanism of GAS6-AS2 in AS remain unclear.

**Aim:**

To investigate the diagnostic/prognostic value of GAS6-AS2 in AS and clarify its molecular mechanism.

**Methods:**

107 AS patients and 105 healthy controls were included. The levels of GAS6-AS2, miR-138-5p, and mRNA were measured using RT-qPCR. ROC curve, K-M survival analysis, and Cox regression were performed to evaluate the diagnostic and prognostic value of GAS6-AS2. Bioinformatics prediction and dual-luciferase reporter assay were performed to verify the regulatory axis. Ox-LDL-induced VSMCs were used to construct an AS cell model. The biological functions were assessed using CCK-8, SA-β-Gal, and ELISA.

**Results:**

GAS6-AS2 expression was significantly increased in AS patients and in VSMCs treated with ox-LDL, and it showed high diagnostic accuracy and risk prediction for patients with AS. Knockdown of GAS6-AS2 reduced SA-β-Gal-positive cells, downregulated the expression of senescence-related genes and proteins (p16, p21, p53), and decreased the levels of inflammatory factors (IL-6, IL-1β) in ox-LDL-induced VSMCs. Mechanistically, GAS6-AS2 directly bound to miR-138-5p and inhibited its expression, while miR-138-5p targeted AKT1 to suppress its expression. Rescue experiments confirmed that the GAS6-AS2/miR-138-5p/AKT1 axis mediated ox-LDL-induced VSMC senescence and inflammation.

**Conclusions:**

GAS6-AS2 is a potential diagnostic and prognostic biomarker for AS. It regulates ox-LDL-induced VSMC senescence and inflammatory response through the sponging of miR-138-5p to upregulate AKT1, providing a novel molecular target for AS treatment.

**Supplementary Information:**

The online version contains supplementary material available at 10.1186/s41065-026-00650-5.

## Background

Atherosclerosis (AS) is defined by having lipid-rich plaques within the arterial wall, and it is counted among the major causes of death around the world [1]. In the later stage of disease progression, plaque rupture can lead to the occurrence of cardiovascular events [2]. Although traditional interventions with lipid-lowering drugs and antiplatelet drugs can delay lipid deposition, some patients still face the threats of plaque progression and acute cardiovascular events [3]. This suggests that delving deeply into the molecular regulatory mechanisms involved in AS pathogenesis is required. The pathological process of AS involves dynamic interactions among multiple cell types, and VSMCs have been identified as the most abundant cell type present in the atherosclerotic plaque [4]. Senescent VSMCs have been observed in both aged blood vessels and atherosclerotic plaques [5], and these senescent cells can promote the development of AS and the formation of plaque vulnerability [6]. Therefore, clarifying the molecular regulatory mechanisms underlying VSMC senescence holds crucial significance for halting the progression of AS.

Long non-coding RNAs (lncRNAs) are characterized by high tissue/cell specificity and considerable stability [7]. They serve as pivotal regulators within the gene regulatory network of AS, including regulating senescence phenotypes and modulating transcription or post-transcriptional regulatory processes [8–10]. For instance, knockdown of lnc-KCNC3-3:1 alleviates the progression of AS by downregulating the JAK1/STAT3 signaling pathway [11]. The lncRNA RP4-639F20.1 exerts a protective effect against AS development by inhibiting VSMCs’ proliferation and migration [12]. LncRNA-MEG3 modulates the miR-26a/Smad1, thereby further regulating the functional balance of VSMCs in AS [13]. Notably, most of these aforementioned lncRNAs have been primarily investigated for their roles in VSMC proliferation and migration, whereas their regulatory roles in cellular senescence have received relatively limited attention. Moreover, RNA sequencing has identified that lncRNA GAS6-AS2 (GAS6-AS2) expression is upregulated during VSMC differentiation [14]. Whether GAS6-AS2 participates in the AS and the manner in which it regulates vascular cell functions via the ceRNA mechanism remains unreported.

Based on this, we analyzed the expression characteristics and diagnostic value of GAS6-AS2 in patients with AS. Then, through bioinformatics, we predicted the downstream-regulated miRNAs and target genes, and constructed a potential ceRNA regulatory axis. Finally, in the VSMCs injury model induced by ox-LDL (a key pathogenic factor of AS), we verified the regulatory mechanism of the “GAS6-AS2/miRNA/gene axis, and its impact on aging, providing a novel theoretical foundation to support the future treatment of AS.

## Materials and methods

### Research objects and sample collection

According to the guidelines for the management of coronary artery syndrome [15], 107 patients with AS who visited the hospital from June 2019 to June 2024 were selected. At the same time, 105 healthy controls (HC) were included. The study group: subjects confirmed by angiography to have arterial wall thickening or complete plaques on the intimal surface. The healthy group: subjects with no family genetic history, no history of diabetes, and normal biochemical indicators. Exclusion criteria: diabetes, malignant tumors, severe hepatic and renal insufficiency, autoimmune diseases, recent infections, history of severe trauma or surgery, and history of drug treatment. Basic information on all study subjects was collected. Total cholesterol (TC), triglyceride (TG), high-density lipoprotein cholesterol (HDL-C), low-density lipoprotein cholesterol (LDL-C), systolic blood pressure (SBP), diastolic blood pressure (DBP), and C-reactive protein (CRP) were routinely detected. Additionally, the IE33 Color Doppler Ultrasonic Imaging instrument (Philips, the Netherlands) was used to examine the carotid intima-media thickness (CIMT). A 60-month follow-up was conducted through outpatient visits or telephone calls to record the occurrence of major adverse cardiovascular events (MACE), which were followed up every three months and included myocardial infarction, ischemic stroke, and cardiovascular death. The ethics committee of the Guanghan People’s Hospital granted approval for this experiment (approval number: 2019 − 0612), with all participants providing their signatures on informed consent forms.

Immediately after admission, venous blood was collected from the elbow vein of each patient. The sample was centrifuged for 15 min at 4 °C. The serum supernatant was collected into a 1.5 milliliter EP tube and stored in a − 80 °C refrigerator.

### Bioinformatics analysis

The miRNAs that bind to GAS6-AS2 were predicted through the lncRNASNP (https://guolab.wchscu.cn/lncRNASNP/) databases. Target genes of the candidate miRNAs can be predicted using starBase. In the meantime, the keyword “atherosclerosis” should be searched in the GeneCards database (https://www.genecards.org/) to acquire the gene set associated with AS. To analyze the target gene set, apply the online platform tool (https://www.bioinformatics.com.cn) to implement both GO enrichment analysis and KEGG pathway analysis.

### Cell culture and construction of ox-LDL injury model

VSMCs were purchased from the Cell Bank of the Chinese Academy of Sciences. The cells were cultured in VSMCs-specific medium (Gibco, with smooth muscle cell growth factor) in an incubator at 37 °C with 5% CO₂. VSMCs were seeded into 96-well plates at a density of 1 × 10⁴ cells/well. After 24 h of culture, the medium was replaced with medium containing different concentrations (0, 20, 40, and 80 µg/mL) of ox-LDL (Sigma-Aldrich), and the cells were further cultured for an additional 24 h.

### RT-qPCR

The serum and total cellular RNA were extracted using TRIzol reagent. For reverse transcription, the PrimeScript RT kit (TaKaRa) was employed. All samples had a 260/280 absorbance ratio around 2.1 and an RNA integrity number ≥ 6.5. The RT-qPCR was performed using SYBR Green Master Mix (Roche). GAPDH was employed as the internal reference for GAS6-AS2 and mRNA analysis, and U6 was used as the internal reference in miRNA analysis. The relative expression level was calculated using the 2^−ΔΔCt^ method. The primer sequences are listed in Table 1.

**Table 1:** The primers used for RT-qPCR

Name		Primer (5‘-3’)
GAS6-AS2	Forward	AAGGAGGACGCAATACC
	Reverse	ATCCTGGCTAACACGGT
miR-138-5p	Forward	GCTTAAGGCACGCGG
	Reverse	GTGCAGGGTCCGAGG
*AKT1*	Forward	CACACCCAGTTCCTGCCT
	Reverse	TCAGATCTGCCCCCATGAGA
*P16*	Forward	CTCGTGCTGATGCTACTGAGGA
	Reverse	GGTCGGCGCAGTT GGGCTCC
*P21*	Forward	GACACCACTGGAGGGTGACT
	Reverse	CAGGTCCACATGGTCT TCCT
*P53*	Forward	GCCATCAACAGCACACA
	Reverse	CCCTTTTCGGAGATTCT
*U6*	Forward	CTCCGCTTCGGCAGCACA
	Reverse	AACGCTTCACGAATTTGCGT
*GAPDH*	Forward	GACTCATGACCACAGTCCATGC
	Reverse	AGAGGCAGGATGATGTTCTG

### Cell transfection

Small interfering RNA targeting GAS6-AS2 (si-GAS6-AS2), miR-138-5p mimics/inhibitors (anti-miR-138-5p), AKT1 overexpression vector (oe-AKT1), and their corresponding negative controls were purchased from GenePharma. According to the instructions of Lipofectamine 3000 (Invitrogen), they were transfected into VSMCs, respectively. After 48 h, the transfection efficiency was detected, and then subsequent experiments were carried out.

### Cell viability assay

The treated VSMCs were seeded in a 96-well cell culture at 1 × 10^4^ cells/well. The cell viability was estimated with a cell counting kit-8 (Dojindo). CCK-8 solution (10 µL/well) was added, and the plates were incubated for 2 h at 37 °C. Subsequently, the absorbance of each well at 450 nm was measured using a microplate reader. The cell viability was presented as a percentage relative to the control.

### Cell senescence (SA-β-Gal)

After washing the cells with PBS, VSMCs were stained using the SA-β-Gal Cellular Senescence Fluorometric Assay Kit (Elabscience) following the manufacturer’s instructions. Cells were analyzed using a BD FACS Calibur flow cytometer.

### Dual-luciferase assay (DLR)

Luciferase reporter plasmids (Yeason) for GAS6-AS2, as well as those for the 3′-untranslated region (3′-UTR) of AKT1 containing wild-type (WT) or mutant (MUT) miR-138-5p binding site sequences (GAS6-AS2-WT, GAS6-AS2-MUT, AKT1-WT, AKT1-MUT), were constructed. Subsequently, we co-transfected the luciferase reporter vectors with miR-138-5p mimics or their negative control (miR-NC) into cells. After 48 h, we used the DLR Gene Assay Kit (Yeason) to detect luciferase activity.

### ELISA

The cell culture supernatant of VSMCs was first collected, and subsequent determination of interleukin-6 (IL-6) and interleukin-1β (IL-1β) levels was conducted by adhering to the operational guidelines of the relevant commercial ELISA kits (Abcam).

### Western blot analysis

The protein was extracted using lysis buffer, and protein concentration was measured via BCA assay kits (Thermo Fisher Scientific). After electrophoresis and membrane transfer, the bands were incubated with the primary antibody at 4 °C overnight, then incubated with the secondary antibody at room temperature for 2 h. Wash the membrane with TBST, develop with ECL, and quantify the gray value of the bands using ImageJ software.

### Data analysis

All experiments were independently repeated 3 times with biological replicates and 3 technical replicates per group. The data are presented as mean ± standard deviation (x ± s). The Shapiro–Wilk test was used to verify the normality of data distribution. An independent-samples t-test was used for comparisons between two groups, and one-way or two-way analysis of variance (ANOVA) was used for comparisons among multiple groups. A *p* < 0.05 was considered statistically significant. GraphPad Prism 10.0 was used for plotting, and SPSS 23.0 was used for statistical analysis.

## Results

### Expression characteristics, diagnostic and prognostic value of GAS6-AS2 in patients with AS

**Fig. 1:**
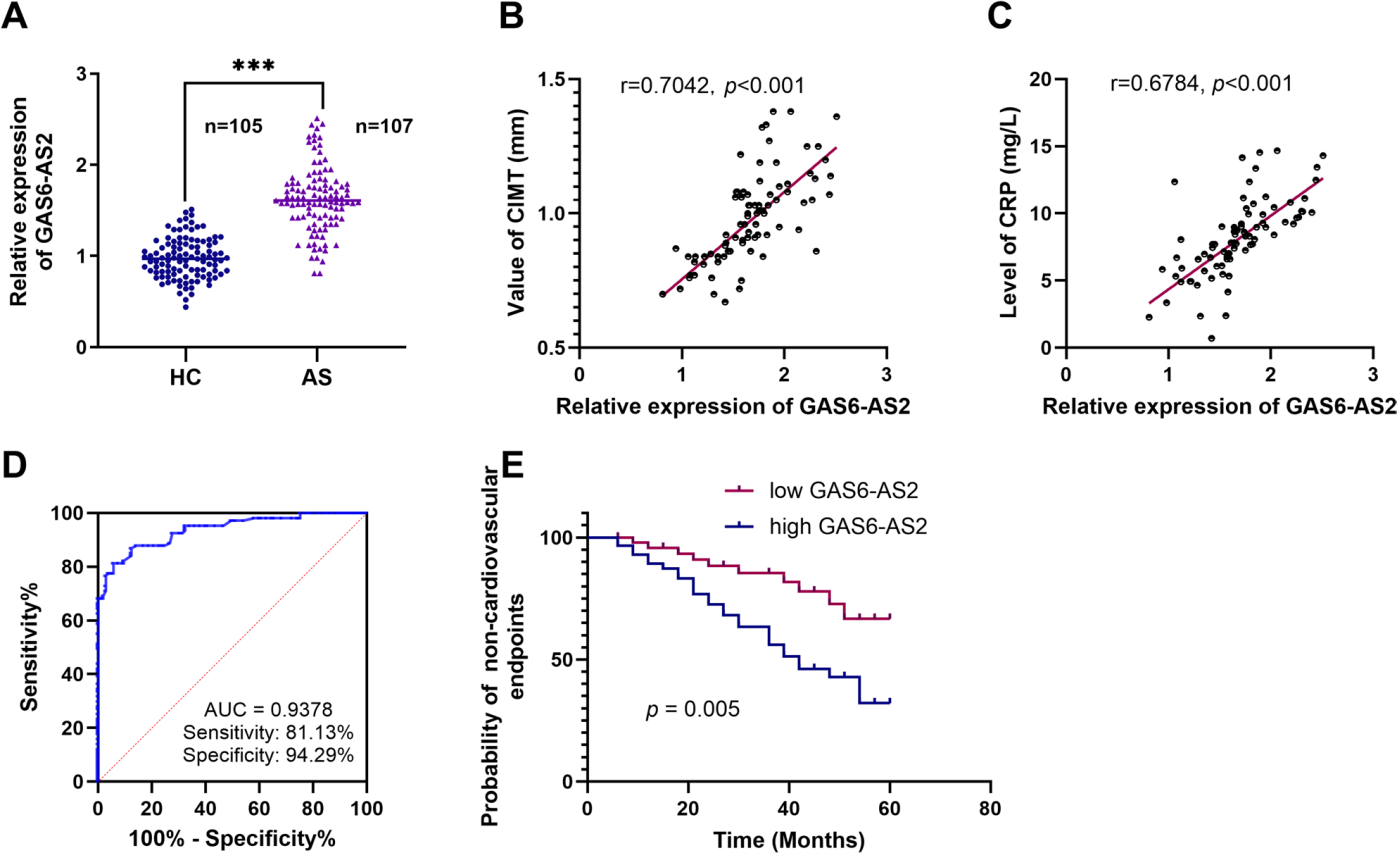
Expression characteristics, diagnostic value, and prognostic significance of GAS6-AS2 in AS patients. **A** RT-qPCR detected the expression levels of GAS6-AS2 in AS patients (n = 107) and healthy controls (HC, n = 105). GAS6-AS2 expression was positively correlated with CIMT (**B**) and CRP (**C**) in the serum of AS patients. **D** ROC showed the efficiency of GAS6-AS2 in diagnosing AS. **E** K-M survival curve showed the relationship between the risk of MACE and the high or low expression of GAS6-AS2 in patients. ***p<0.001

To verify the association between GAS6-AS2 and AS, 107 patients with diagnosed AS and 105 HC were enrolled. RT-qPCR analysis revealed that the serum GAS6-AS2 expression in AS patients was notably higher compared to that in HC (Fig. 1A), suggesting that GAS6-AS2 may be associated with the occurrence of AS. When comparing baseline data between the AS group and the HC group, no significant differences were observed in age, gender, BMI, drinking history, smoking history, TC, HDL-C, TG, SBP, and DBP between the two groups, while LDL-C, CRP, and CIMT showed significant increases (Table 2). Correlation analysis revealed a significant positive correlation between the serum expression level of GAS6-AS2 and CIMT thickness (*r* = 0.7042, *p* < 0.001, Fig. 1B), as well as with the content of CRP (*r* = 0.6784, *p <* 0.001, Fig. 1C), suggesting that GAS6-AS2 may be associated with the inflammatory response of AS and the risk of cardiovascular disease. Based on the expression levels of GAS6-AS2 in the AS group and the HC group, receiver operating characteristic (ROC) curve analysis showed that the area under the ROC curve (AUC) of GAS6-AS2 was 0.9378 (95% CI: 0.9065–0.9690), and its sensitivity was 81.13% and the specificity was 94.29% (Fig. 1D), suggesting that GAS6-AS2 has a high diagnostic value for AS. With MACE as the dependent variable and the average expression level of GAS6-AS2 included to stratify groups, Kaplan-Meier (K-M) curve results showed that high expression of GAS6-AS2 was significantly associated with an increased risk of MACE (Fig. 1E). In addition, the results of multifactorial Cox analysis showed that GAS6-AS2 (HR = 6.390, *p* = 0.028), CRP (HR = 12.638, *p* = 0.001), and CIMT (HR = 3.134, *p* = 0.021) were risk factors for the occurrence of AS (Table 3).

**Table 2:** Clinical characteristics of the study subjects

Characteristics	HC (*n* = 105)	AS (*n* = 107)	*p*
Age (years)	60.33 ± 8.81	57.80 ± 11.91	0.081
Gender (male/female)	47 / 58	51 / 56	0.674
Drinking	51 / 59	57 / 50	0.399
Smoking	17 / 88	18 / 89	0.901
BMI (kg/m2)	22.40 ± 2.45	21.84 ± 2.28	0.808
TC (mg/dL)	184.53 ± 24.59	179.45 ± 24.19	0.132
HDL-C (mg/dL)	45.95 ± 9.10	48.91 ± 25.19	0.267
LDL-C (mg/dL)	115.76 ± 15.33	122.78 ± 14.59	0.001
TG (mg/dL)	121.31 ± 24.90	121.42 ± 24.21	0.202
DBP (mm Hg)	79.36 ± 10.39	80.34 ± 9.15	0.466
SBP (mm Hg)	128.36 ± 14.91	129.95 ± 9.70	0.356
CRP (mg/L)	1.97 ± 0.54	8.69 ± 2.84	< 0.001
CIMT (mm)	0.58 ± 0.18	1.01 ± 0.16	< 0.001

Data are presented as mean ± SD

*HC* Healthy controls, *AS* Atherosclerosis, *TC* Total cholesterol, *HDL-C* High-density lipoprotein cholesterol, *LDL-C* Low-density lipoprotein cholesterol, *TG* Triglyceride, *SBP* Systolic blood pressure, *DBP* Diastolic blood pressure, *CRP* C-reactive protein, *CIMT* Carotid Intima-Media Thickness

***p < 0.001

**Table 3:** Multifactorial Cox analysis

Characteristics	HR	95.0% CI for HR		*p*
		Lower	Upper	
GAS6 - AS2	6.390	1.221	33.428	0.028
Age	1.451	0.644	3.269	0.369
Gender	1.211	0.549	2.674	0.635
Drinking	1.102	0.522	2.326	0.798
Smoking	1.851	0.734	4.673	0.192
BMI	1.199	0.568	2.528	0.634
TC	1.106	0.364	3.361	0.858
HDLC	1.462	0.376	5.691	0.583
LDLC	1.325	0.502	3.495	0.570
TG	2.112	0.926	4.818	0.075
DBP	1.584	0.724	3.465	0.250
SBP	1.184	0.537	2.609	0.675
CRP	12.638	2.707	59.012	0.001
CIMT	3.134	1.189	8.259	0.021

*TC* Total cholesterol, *HDL-C* High-density lipoprotein cholesterol, *LDL-C* Low-density lipoprotein cholesterol, *TG* Triglyceride, *SBP* Systolic blood pressure, *DBP* Diastolic blood pressure, *CRP* C-reactive protein, *CIMT* Carotid Intima-Media Thickness

***p < 0.001

### The reversal effect of GAS6-AS2 Silencing on ox-LDL-induced VSMC injury

**Fig. 2:**
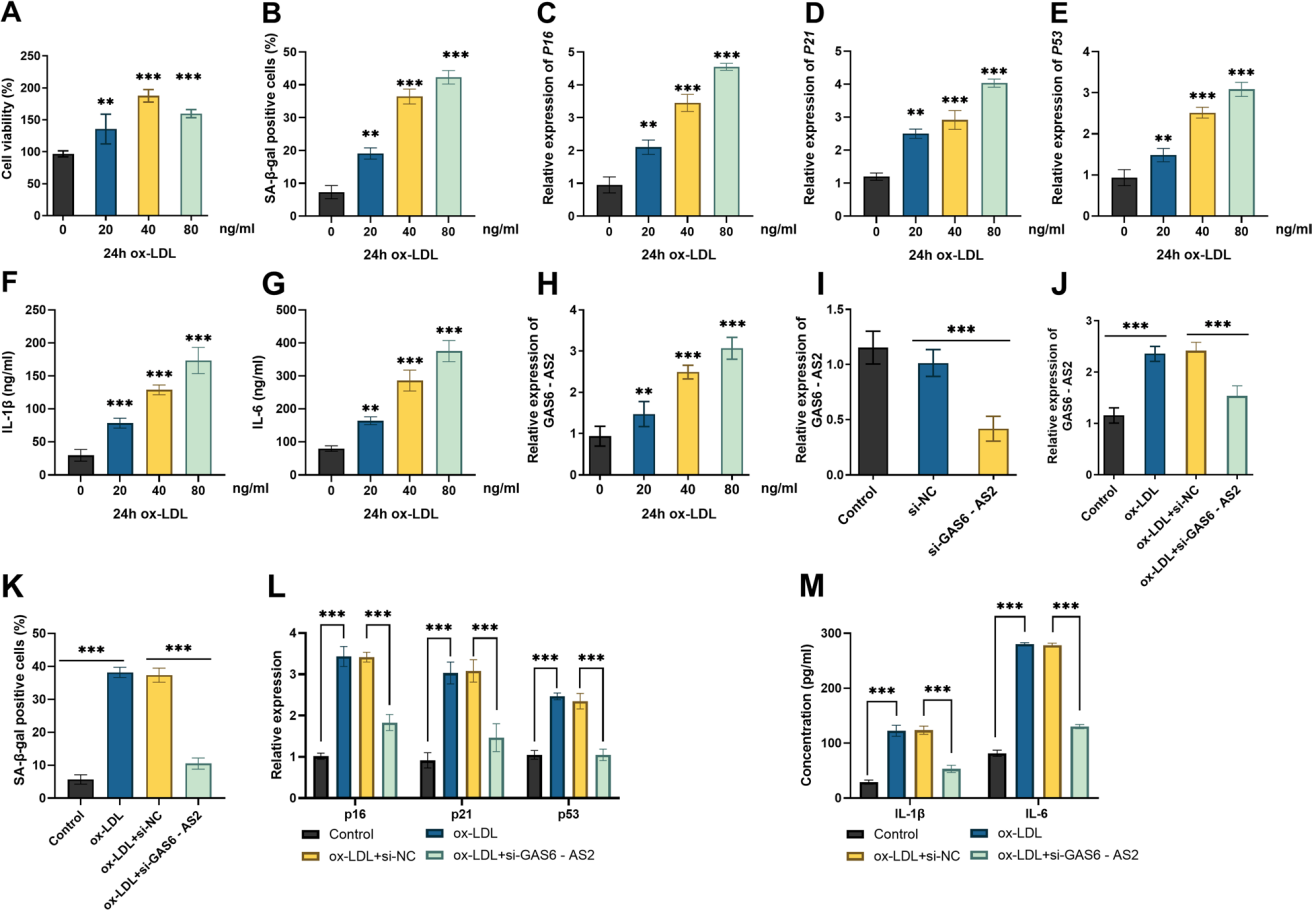
The reversing effect of GAS6-AS2 knockdown on the injury of VSMCs induced by ox-LDL. After treating VSMCs with different concentrations of ox-LDL (0, 20, 40, 80 µg/mL) for 24 h, the cell viability (**A**), SA-β-Gal positive cells (**B**), the levels of senescence-related genes p16 (**C**), p21 (**D**), p53 (**E**), and inflammatory factors IL-1β (**F**), IL-6 (**G**) were measured. **H** The expression level of GAS6-AS2 in VSMCs after treatment with different concentrations of ox-LDL for 24 h. **I**-**J** The expression level of GAS6-AS2 after transfection with si-GAS6-AS2 in VSMCs or ox-LDL-induced VSMCs. After knockdown of GAS6-AS2, the proportion of SA-β-Gal positive cells (**K**), the levels of senescence-related genes p16, p21, p53 (**L**), and inflammatory factors IL-6, IL-1β (**M**) in ox-LDL-treated VSMCs. **p < 0.01, ***p < 0.001

When the AS cellular model was treated with ox-LDL, cell viability showed a dose-dependent increase at concentrations below 40 µg/mL, but decreased when the concentration reached 80 µg/mL (Fig. 2A), and a dose-dependent promotion of SA-β-Gal-positive cells (Fig. 2B) was observed. Concurrently, the expression of senescence-associated genes p16, p21, and p53 was upregulated (Fig. 2C-E), and the levels of inflammatory mediators IL-1β and IL-6 were increased (Fig. 2F-G). Since significant changes in all these indicators occurred under the stimulation of 40 µg/mL ox-LDL and in the context of cell senescence, 40 µg/mL ox-LDL was used for subsequent analysis. After exposure to different concentrations of ox-LDL in VSMCs, the expression of GAS6-AS2 was upregulated in a dose-dependent manner (Fig. 2H). Subsequently, transfection with si-GAS6-AS2 in VSMC significantly decreased the level of GAS6-AS2 (Fig. 2I). The upregulated effect of ox-LDL treatment on GAS6-AS2 expression was inhibited by si-GAS6-AS2 (Fig. 2J). We observed that si-GAS6-AS2 reduced the number of SA-β-Gal-positive cells (Fig. 2K), downregulated the expression of p16, p21, and p53 (Fig. 2L), and decreased the levels of IL-6 and IL-1β (Fig. 2M). The above results indicate that knockdown of GAS6-AS2 can reverse the damage to VSMCs treated with ox-LDL.

### Bioinformatics prediction of the downstream regulatory network of GAS6-AS2

**Fig. 3:**
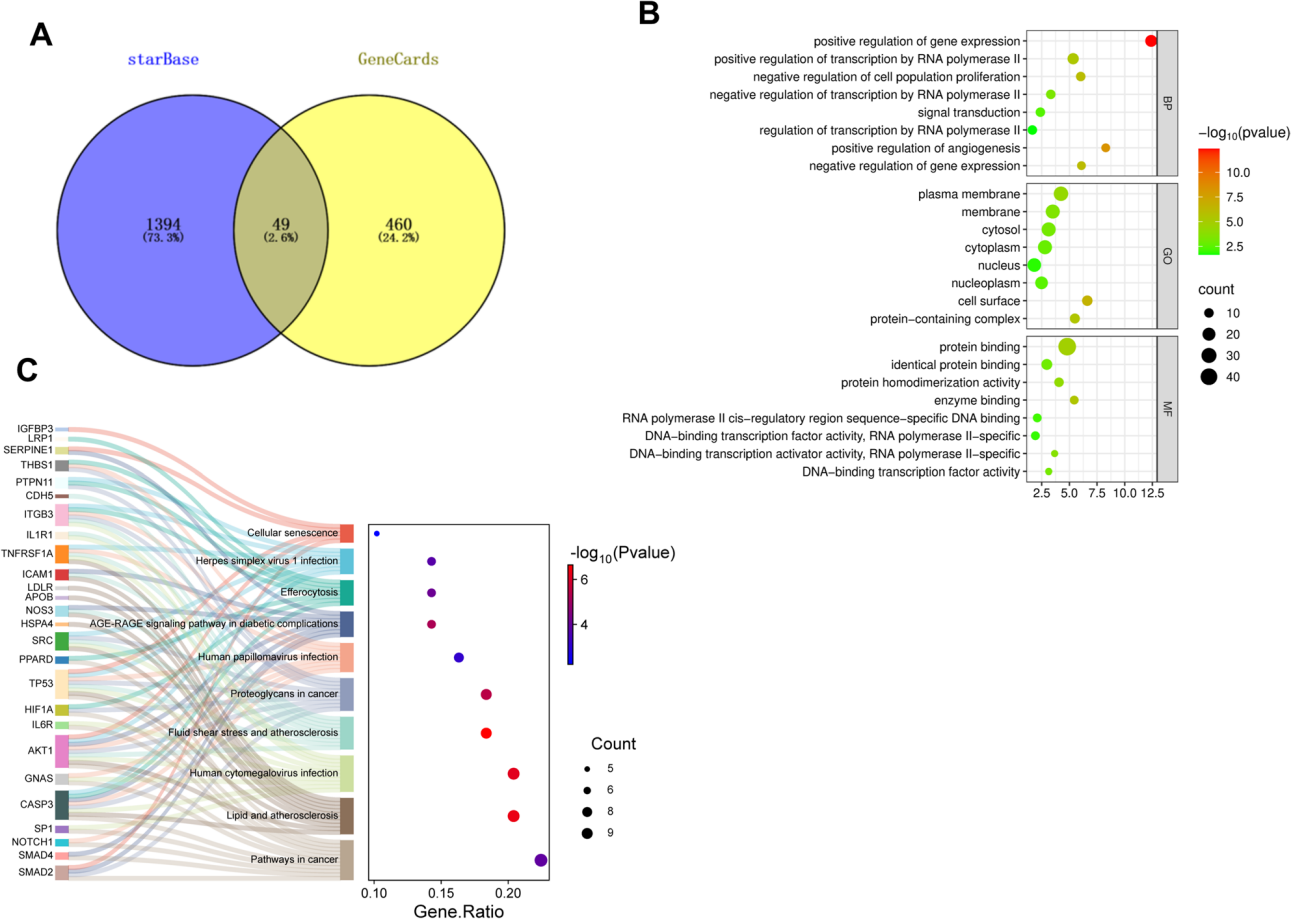
Bioinformatics prediction and analysis of the downstream regulatory network of GAS6-AS2. **A** miR-138-5p target genes predicted via the starbase database, intersected with AS-related genes from GeneCard. GO (**B**) and KEGG (**C**) enrichment analysis for intersection genes

The downstream miRNAs of GAS6-AS2 were predicted through the lncRNASNP databases. Among them, miR-138-5p has been reported to be related to AS. Furthermore, the downstream targets of miR-138-5p were predicted through the starbase database and intersected with the genes related to AS in the GeneCard database, and 49 genes were screened out (Fig. 3A). After that, we performed GO and KEGG analyses on these 49 genes (Fig. 3B). Biological Process (BP) analysis indicated that genes were significantly involved in the regulation of expression and transcription. Cellular Component (CC) analysis indicated that genes were enriched in subcellular structures, including the plasma membrane, cytoplasm, and nucleus. Molecular Function (MF) analysis results illustrated that there was a high degree of enrichment of genes in molecular functions; typical examples of these functions are protein–protein interaction and the DNA-binding activity of transcription factors. KEGG analysis revealed that common genes were implicated in lipid and atherosclerosis, fluid shear stress, and cellular senescence (Fig. 3C). Among them, AKT1 is jointly involved in these three pathways.

### The targeted binding relationship between GAS6-AS2 and miR-138-5p

**Fig. 4:**
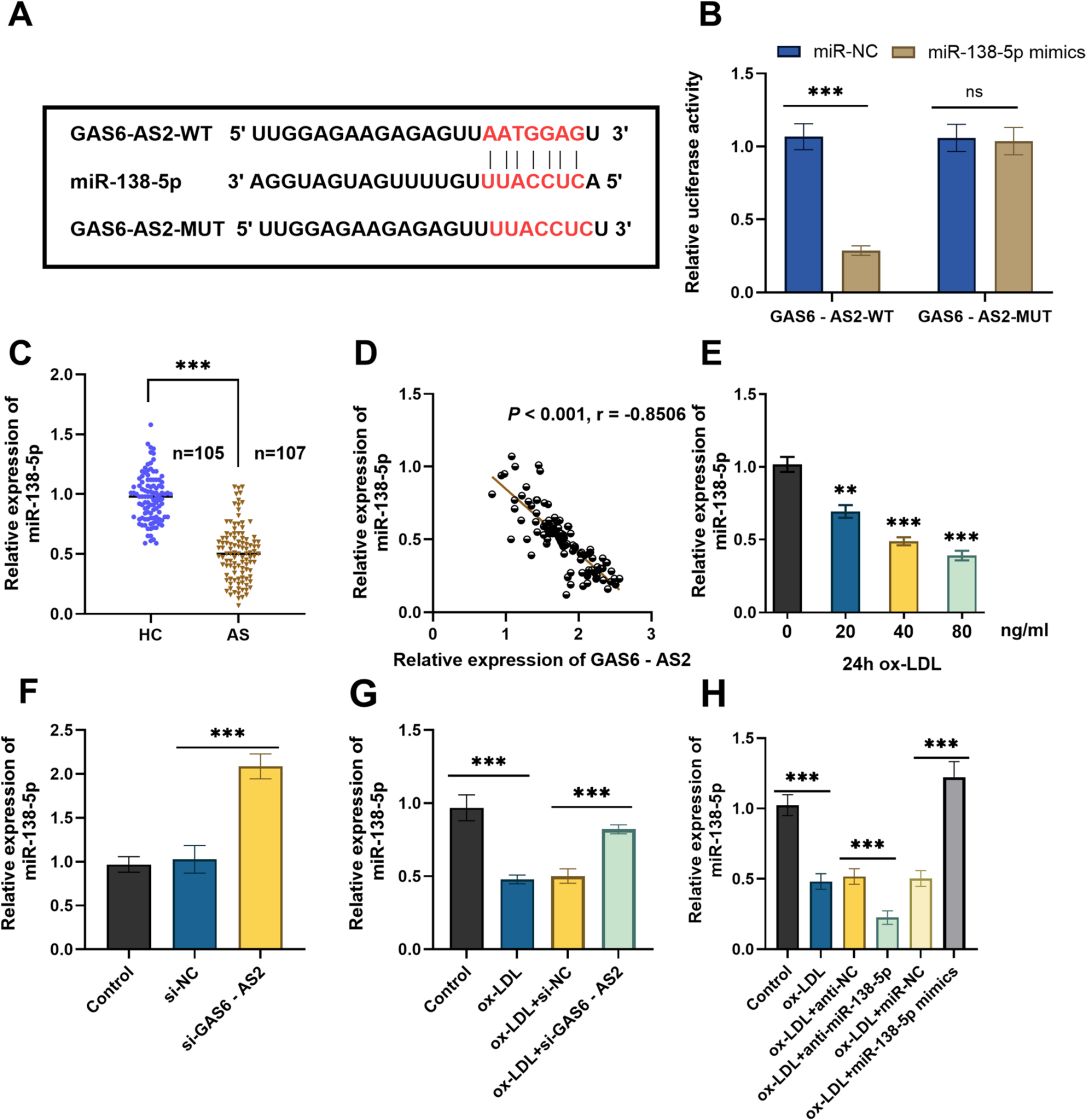
Validation of the targeted binding between GAS6-AS2 and miR-138-5p. **A** Potential binding sites between GAS6-AS2 and miR-138-5p. **B** A dual-luciferase reporter assay was performed to detect the luciferase activity of wild-type and mutant GAS6-AS2 reporter genes after transfection with miR-138-5p mimics in VSMCs. **C** RT-qPCR was used to detect the expression of miR-138-5p in the serum of AS patients and HC. **D** GAS6-AS2 expression was negatively correlated with miR-138-5p in the serum of AS patients. **E** Expression of miR-138-5p after treatment of VSMCs with different concentrations of ox-LDL. **F**-**G** The expression level of miR-138-5p after transfection with si-GAS6-AS2 in VSMCs or ox-LDL-induced VSMCs. **H** RT-qPCR verified the expression efficiency of miR-138-5p mimics and anti-miR-138-5p. ns, no statistical difference. **p < 0.01, ***p < 0.001

According to the prediction results, GAS6-AS2 was predicted to contain a binding site for miR-138-5p (Fig. 4A). DLR assay showed that in VSMCs, the miR-138-5p mimic significantly reduced the luciferase activity of the GAS6-AS2-WT, but had no such effect on the GAS6-AS2-MUT (Fig. 4B). RT-qPCR results showed that the serum expression level of miR-138-5p in AS patients was significantly lower than that in HC (Fig. 4C), and it was negatively correlated with the expression of GAS6-AS2 (Fig. 4D). In addition, ox-LDL treatment decreased the expression level of miR-138-5p in VSMCs in a dose-dependent manner (Fig. 4E). In VSMC, si-GAS6-AS2 increased the expression of miR-138-5p (Fig. 4F). The decreased effect of ox-LDL treatment VSMC on miR-138-5p expression was reversed by si-GAS6-AS2 (Fig. 4G). The expression efficiency of the miR-138-5p mimic and inhibitor in ox-LDL-induced VSMCs was confirmed (Fig. 4H). Overall, GAS6-AS2 directly binds to miR-138-5p and inhibits its expression in VSMCs.

### The targeted verification of miR-138-5p and AKT1 and the GAS6-AS2/miR-138-5p/AKT1 regulatory circuit

**Fig. 5:**
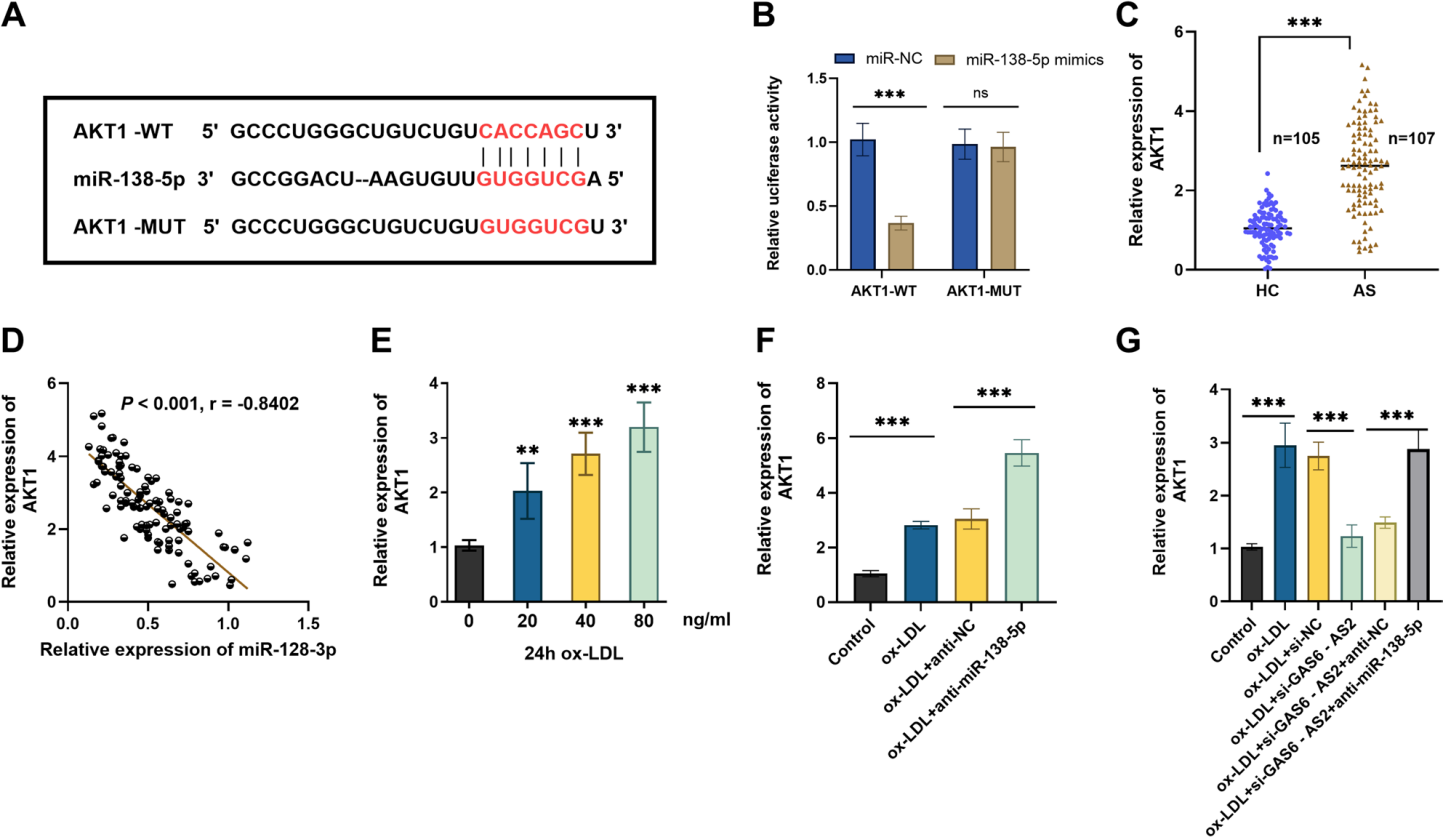
Targeted verification of miR-138-5p and AKT1. **A** Potential binding sites between the AKT1 and miR-138-5p. **B** A dual-luciferase reporter gene assay was performed to detect the luciferase activities of wild-type and mutant AKT1 reporter genes after transfection of miR-138-5p in VSMCs. **C** RT-qPCR detected the expression of AKT1 in the serum of AS patients and HC. **D** The expression of miR-138-5p and AKT1 in the serum of AS patients was negatively correlated. **E** Expression levels of AKT1 after treatment of VSMCs with different concentrations of ox-LDL. **F** Expression of AKT1 in ox-LDL-induced VSMCs transfected with anti-miR-138-5p. **G** Expression of AKT1 in ox-LDL-treated VSMCs co-transfected with si-GAS6-AS2 and anti-miR-138-5p. **p < 0.01, ***p < 0.001

There is a targeted binding site between AKT1 and miR-138-5p (Fig. 5A). The results of the DLR assay showed that in VSMCs, the miR-138-5p mimics significantly reduced the luciferase activity of the AKT1*-WT* luciferase reporter gene, but had no such effect on AKT1*-MUT* (Fig. 5B). RT-qPCR analysis revealed that the expression level of AKT1 in the serum samples from AS patients was remarkably higher compared to that in HC (Fig. 5C), and there was a negative correlation between it and the expression level of miR-138-5p (Fig. 5D). In addition, ox-LDL treatment increased the expression level of AKT1 in VSMCs in a dose-dependent manner (Fig. 5E). It was observed that the expression of AKT1 in VSMCs was increased due to the inhibition of miR-138-5p (Fig. 5F). Therefore, we confirmed that miR-138-5p targeted and inhibited the expression of AKT1 in VSMCs. Considering that GAS6-AS2 can adsorb miR-138-5p, and miR-138-5p targets AKT1, we subsequently co-transfected si-GAS6-AS2 and anti-miR-138-5p. The results proved that in ox-LDL-treated VSMCs, knockdown of GAS6-AS2 led to a decrease in AKT1 expression, and subsequent transfection of the miR-138-5p inhibitor could rescue this decrease (Fig. 5G), which revealed the regulatory effect of GAS6-AS2/miR-138-5p on AKT1 in VSMCs.

### The function of the GAS6-AS2/miR-138-5p/AKT1 axis in ox-LDL-induced VSMC injury

**Fig. 6:**
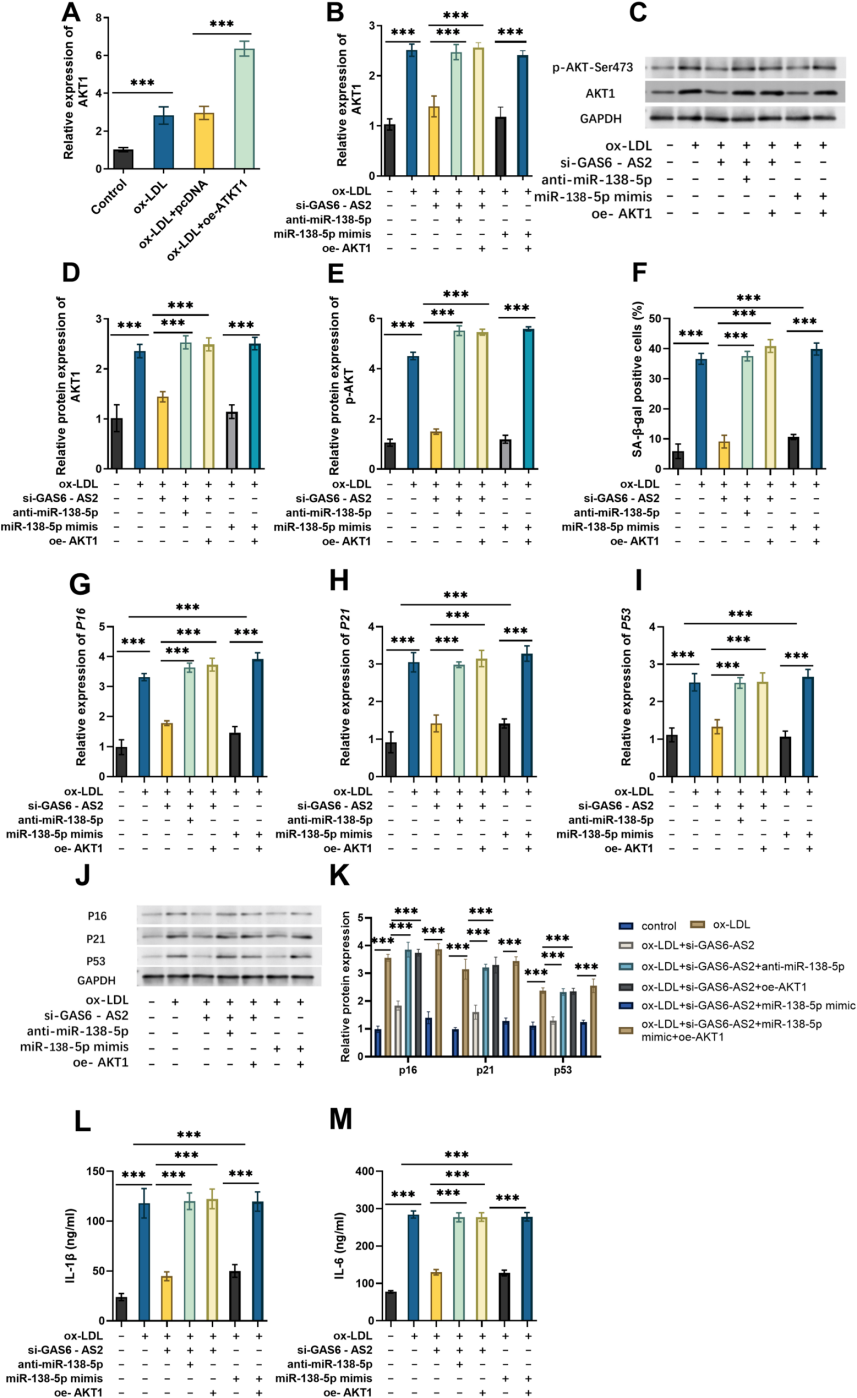
Functional verification of the GAS6-AS2/miR-138-5p/AKT1 axis in the injury of VSMCs induced by ox-LDL. **A** Expression of AKT1 in ox-LDL-induced VSMCs transfected with the oe-AKT1. Effects of transfecting si-GAS6-AS2, miR-138-5p mimics, anti-miR-138-5p, and oe-AKT1 in ox-LDL-induced VSMCs on AKT1 mRNA level, AKT1 and p-AKT protein levels (**C**-**E**), the proportion of SA-β-Gal positive cells (**F**), and the levels of senescence-related genes p16 (**G**), p21 (**H**), p53 (**I**), the protein levels of p16, p21, p53 (**J**-**K**) and inflammatory factors IL-1β (**L**), IL-6 (**M**). ***p < 0.001

Finally, we investigated the role of the GAS6-AS2/miR-138-5p/AKT1 axis in ox-LDL-treated VSMCs, after verifying the expression efficiency of the AKT1 overexpression plasmid in ox-LDL-induced VSMCs (Fig. 6A). Anti-miR-138-5p or oe-AKT1 reversed the inhibitory effects of GAS6-AS2 knockdown on the AKT1expression. MiR-138-5p mimic further decreased the AKT1 expression, and oe-AKT1 reversed its effect (Fig. 6B). The protein expression of AKT1 and p-*AKT* also showed the same trend (Fig. 6C-E). Anti-miR-138-5p or oe-AKT1 reversed the inhibitory effects of GAS6-AS2 knockdown in the SA-β-Gal positive cells, miR-138-5p mimics further decreased the SA-β-Gal positive cells, and oe-AKT1 reversed its effect (Fig. 6F). Anti-miR-138-5p or oe-AKT1 restored the mRNA expression levels of p16, p21, and p53 (Fig. 6G-I) in the VSMCs with GAS6-AS2 silencing. miR-138-5p mimics decreased the levels of senescence-related gene expression and inflammatory factor production, but this effect was reversed by oe-AKT1. The protein expression of p16, p21, and p53 (Fig. 6J-K) and the inflammatory factors (Fig. 6L-M) showed the same trend as mRNA. The above results indicate that GAS6-AS2 can promote ox-LDL-induced cell senescence and enhanced inflammatory response, and its mechanism is achieved by targeting miR-138-5p to regulate AKT1 expression.

## Discussion

AS is essentially an aging-related vascular disorder, and aging itself has been established as an independent risk factor for its development. Accumulating evidence has confirmed that age-associated accumulation of senescent vascular wall cells and exacerbation of an inflammatory microenvironment directly drive the formation and progression of AS plaques [16]. LncRNAs, as a class of non-coding RNAs with diverse regulatory functions, have been identified as key modulators in aging-related disorders and the pathogenesis of AS. For example, lnc-IL7R mediates cellular senescence and apoptosis by inducing oxidative stress [17]. Upregulation of lnc-HRK-2:1 promotes nucleus pulposus cell senescence [18]. LncRNA MEG3 is downregulated in AS patients and mice, with MEG3 deficiency exacerbating cellular senescence [19]. Notably, lncRNA GAS6-AS2 has been implicated in other inflammatory and stress-related pathologies, with its downregulation in acute kidney injury alleviating apoptosis, inflammatory responses, and oxidative stress [20], and mediating inflammatory cascades in periodontitis [21]. In this study, it was observed that the expression level of GAS6-AS2 in the serum of AS patients was significantly elevated. There is a close correlation between the CIMT and the onset of diverse cardiovascular diseases [22]. CRP is an effective predictor and risk factor for cardiovascular disease [23]. Additional analyses revealed that the expression level of GAS6-AS2 was significantly positively associated with CIMT and CRP, which further supports that GAS6-AS2 is involved in regulating the process of AS. The ROC curve showed that GAS6-AS2 had high diagnostic accuracy for AS. KM curve and COX analysis revealed that high expression of GAS6-AS2 was significantly associated with the risk of MACE in AS patients, suggesting that it can act as a potential risk factor and prognostic indicator for major adverse cardiovascular events in AS patients.

In view of the high expression of GAS6-AS2 in the serum of AS patients, we further investigated its expression in damaged cells. As the core constituent cells of the fibrous cap of AS plaques, the abnormal functions (senescence, apoptosis, and inflammatory activation) of VSMCs in these plaques represent the decisive step in the transition of plaques from a stable to a vulnerable phenotype [5, 24]. Treatment with ox-LDL can lead to cellular senescence phenotypes and inflammatory responses [25, 26]. In this study, GAS6-AS2 was highly expressed in Ox-LDL-treated VSMCs. After we knocked out GAS6-AS2, the senescence marker (SA-β-Gal), related genes (p16, p21, p53), and the release of inflammatory factors (IL-1β, IL-6) were significantly downregulated. These indicators, as classic markers for detecting cell senescence, have been widely verified for their reliability [19, 27, 28]. This series of results clearly confirms that the deletion of GAS6-AS2 can effectively suppress the ox-LDL-induced senescent phenotype in VSMCs.

In this study, we employed bioinformatics prediction approaches, and the results indicated that GAS6-AS2 has a potential binding site for miR-138-5p. Previous studies have constructed the functional framework of miR-138-5p in vascular diseases. It is downregulated in ox-LDL-induced endothelial dysfunction models and can promote AS progression to a certain extent [29]. In peripheral coronary artery disease, it acts as a post-transcriptional regulator associated with vascular injury [30]. Meanwhile, low miR-138-5p expression is linked to senescence in multiple cell types, including fibroblasts and osteoblasts [31, 32]. Building on this, the present study further expands and clarifies that GAS6-AS2 directly targets miR-138-5p, and that a significant negative correlative relationship exists between them in AS. It has been verified that miR-138-5p is capable of reversing the effect of GAS6-AS2 on VSMC senescence and inflammation, indicating that the regulation of VSMCs by GAS6-AS2 depends on the adsorption of miR-138-5p.

AKT1 is a classic molecule involved in both inflammatory and aging-related processes. The PI3K/Akt signaling pathway is the most widespread and classic pathway where Akt is involved, and its activation in AS has been widely demonstrated [33]. Inhibiting the AKT1 pathway can effectively block the inflammatory cascade induced by ox-LDL, and this mechanism represents a promising therapeutic strategy for the prevention and treatment of AS [34]. AKT1 has been shown to be involved in regulating VSMC function and inflammatory responses [35]. In this study, AKT1 is a downstream target of miR-138-5p, which is involved in the inflammatory and aging signaling pathways. Experimental verification showed that AKT1 is highly expressed in AS patients and ox-LDL-treated VSMCs, and overexpression of AKT1 exacerbates cellular senescence and inflammatory responses. Meanwhile, in terms of regulatory mechanisms, overexpression of AKT1 can reverse the protective effects of GAS6-AS2 knockout on VSMCs and exacerbate the promoting effect of miR-138-5p on senescence and inflammation, indicating that AKT1 is a core effector molecule of the GAS6-AS2/miR-138-5p axis. AKT1 has been documented to interact with multiple pathways crucial for VSMCs, such as the MAPK [36], mTOR [37], NF-κB [38], and FOXO [39] pathways. In vascular diseases, ERK1/2, p38MAPK, and PKB/Akt signaling are synchronously activated [40]. Future research could clarify the complete signaling cascade linking GAS6-AS2 and VSMC senescence by regulating the expression of GAS6-AS2 or AKT1.

In this study, a few limitations are worth highlighting. First, AS is caused by a more complex interaction between hemodynamic stress, endothelial dysfunction, and chronic inflammation driven by multiple cell types, we only used the ox-LDL-induced injury model of VSMC for our study, so it cannot fully simulate the real state of AS; secondly, our in vitro experiments only verified the intracellular role of GAS6-AS2 in cultured vascular smooth muscle cells, but did not verify its ability to be secreted by cells or taken up by target cells in vivo; finally, the current research only analyzed AKT1, ignoring the potential synergistic effects of other miR-138-5p targets, which may limit the comprehensiveness of the proposed regulatory mechanism.

## Conclusion

This study confirmed that GAS6-AS2 is highly expressed in the serum of AS patients and is a potential diagnostic and prognostic biomarker for AS. It sponges miR-138-5p through the ceRNA mechanism, relieves the inhibition of AKT1, and thus promotes the senescence and inflammatory responses of VSMCs, exacerbating the pathological progression of AS. The GAS6-AS2/miR-138-5p/AKT1 axis provides a new perspective on the pathogenesis of AS.

## Supplementary Information

Supplementary Material 1.

Supplementary Material 2.

## Data Availability

The datasets used and/or analyzed during the current study are available from the corresponding author on reasonable request.
