# Supplementary figures and images for: LncRNA GAS6-AS2 regulates vascular smooth muscle cell senescence through the miR-138-5p/AKT1 axis and serves as a diagnostic and prognostic marker for atherosclerosis
Source: Hereditas. 2026 Feb 4;163:38. doi: 10.1186/s41065-026-00650-5 (PMC12958701; doi:10.1186/s41065-026-00650-5)

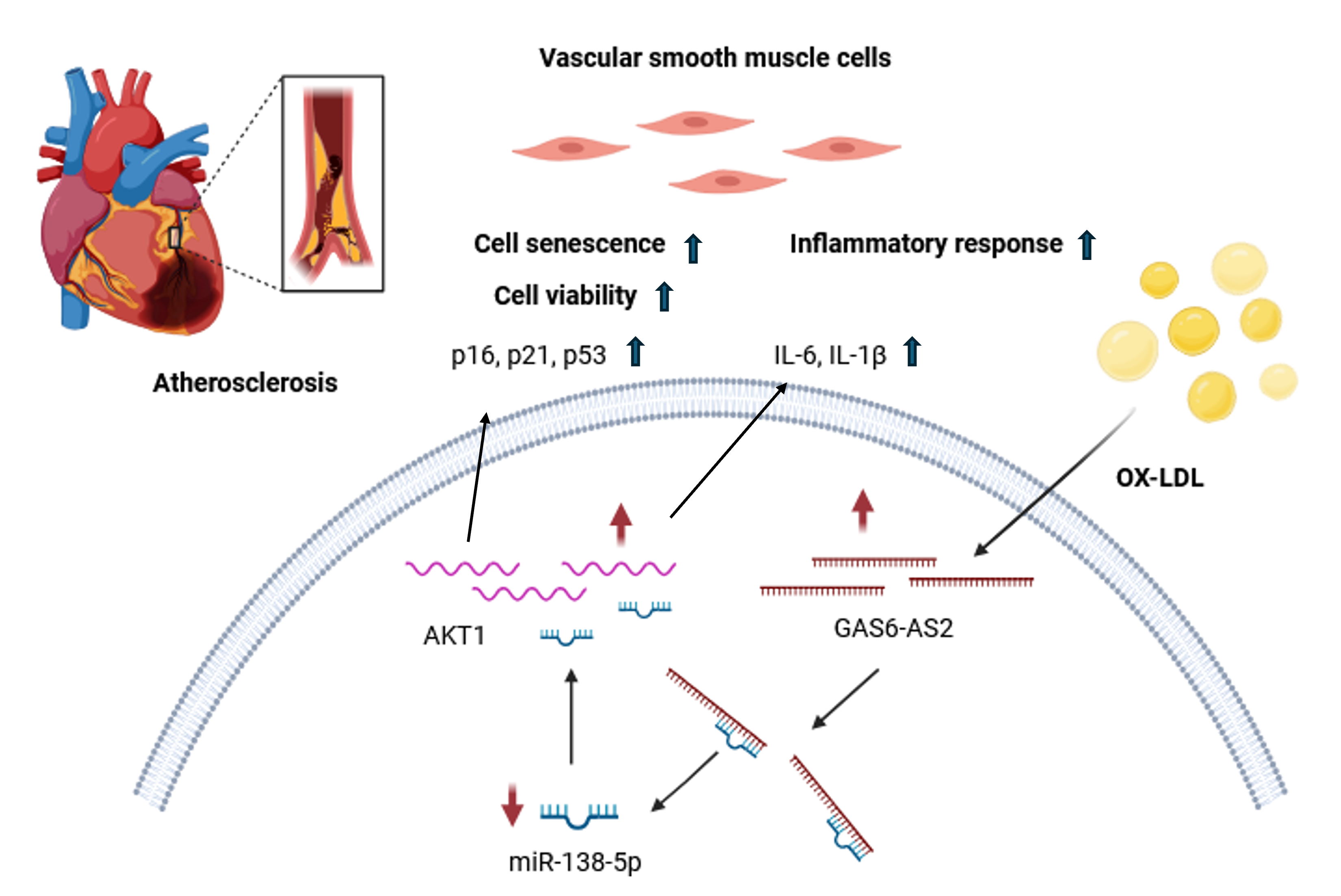

Supplement: Supplementary file 1 — Supplementary Material 1. [file 41065_2026_650_MOESM1_ESM.tif]

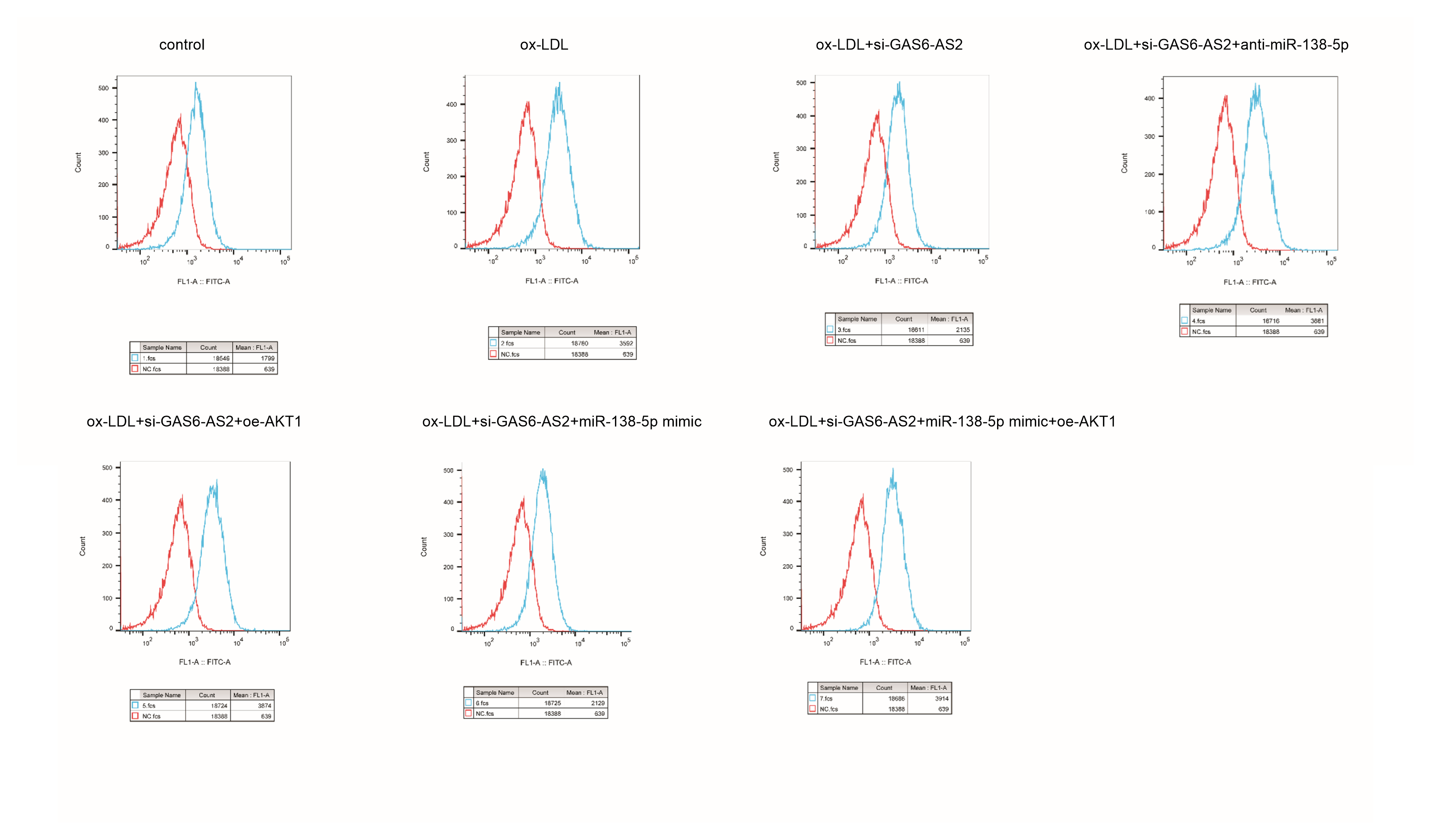

Supplement: Supplementary file 2 — Supplementary Material 2. [file 41065_2026_650_MOESM2_ESM.tif]
